# The Mechanisms of Antibacterial Activity of Magnesium Alloys with Extreme Wettability

**DOI:** 10.3390/ma14185454

**Published:** 2021-09-21

**Authors:** Alexandre M. Emelyanenko, Alexander G. Domantovsky, Valery V. Kaminsky, Ivan S. Pytskii, Kirill A. Emelyanenko, Ludmila B. Boinovich

**Affiliations:** 1 A.N. Frumkin Institute of Physical Chemistry and Electrochemistry, Leninsky Prospect 31 bldg. 4, 119071 Moscow, Russia; ame@phyche.ac.ru (A.M.E.); doman-alex@yandex.ru (A.G.D.); ivanpic4586@gmail.com (I.S.P.); emelyanenko.kirill@gmail.com (K.A.E.); 2 G.N. Gabrichevsky Research Institute for Epidemiology and Microbiology, 10 Admiral Makarov St., 125212 Moscow, Russia; kaminskyvalery86@gmail.com; 3 Russian Scientific Center of Roentgenoradiology, Ministry of Healthcare of the Russian Federation, 117997 Moscow, Russia

**Keywords:** antibacterial surfaces, laser surface modification, bacteriolysis, *Pseudomonas aeruginosa*, *Klebsiella pneumoniae*, superhydrophobicity, superhydrophilicity, bacterial dispersions, bactericidal activity

## Abstract

In this study, we applied the method of nanosecond laser treatment for the fabrication of superhydrophobic and superhydrophilic magnesium-based surfaces with hierarchical roughness when the surface microrelief is evenly decorated by MgO nanoparticles. The comparative to the bare sample behavior of such surfaces with extreme wettability in contact with dispersions of bacteria cells *Pseudomonas aeruginosa* and *Klebsiella pneumoniae* in phosphate buffered saline (PBS) was studied. To characterize the bactericidal activity of magnesium samples with different wettability immersed into a bacterial dispersion, we determined the time variation of the planktonic bacterial titer in the dispersion. To explore the anti-bacterial mechanisms of the magnesium substrates, a set of experimental studies on the evolution of the magnesium ion concentration in liquid, pH of the dispersion medium, surface morphology, composition, and wettability was performed. The obtained data made it possible to reveal two mechanisms that, in combination, play a key role in the bacterial decontamination of the liquid. These are the alkalization of the dispersion medium and the collection of bacterial cells by microrods growing on the surface as a result of the interaction of magnesium with the components of the buffer solution.

## 1. Introduction

The development of antimicrobial resistance of common pathogens is one of the key challenges of modern science and public health. Analyses performed in the literature have shown that antimicrobial resistance will cause about 10 million deaths by 2050 [1,2]. According to the World Health Organization, an estimated 80 percent of infectious diseases are transmitted by touch or through touch surfaces. Therefore, environmentally-friendly approaches leading to the eradication of bacteria on frequently touched surfaces without causing resistance can be considered as a prospective way to combat cross infections. Currently, a biomimetic strategy is being actively developed based on the use of the antibacterial properties of nanoparticles and metal surfaces with hierarchical roughness [3,4,5,6,7,8,9,10]. The antimicrobial mechanisms caused by nanoparticles that were recently discussed in the literature include damage and impairment of the functioning of DNA, protein and cell membrane, cell signaling, and breakdown of cellular structures [10,11,12]. The above processes are either induced or enhanced by the metal ion release and/or excess of different reactive oxygen species, generated by nanoparticles [12,13,14].

The application of magnesium-based nanomaterials for antibacterial purposes is of particular interest. On the one hand, magnesium alloys have been proposed as a new class of metallic bioresorbable and biocompatible cardiovascular stents [15,16], bone or dental replacement implant material [17,18], and catheters [19], with a low or a mild level of cytotoxicity with respect to mammalian cells [18,20]. On the other hand, recent studies consider magnesium-containing nanoparticles and nanoparticle-decorated surfaces as demonstrating a bactericidal effect against many widespread pathogens [20,21,22,23,24,25,26,27].

The antibacterial activity of magnesium oxide nanoparticles was studied in various systems [21,22,23,24,25,26]. MgO nanoparticles electrophoretically deposited onto poly-L-lactic acid sheets have shown the ability to resist colonization by *Staphylococcus aureus*, *Staphylococcus epidermidis*, and *Pseudomonas aeruginosa* with the simultaneous promotion of bone cell attachment and curb fibrous capsule formation [21]. The strong bactericidal activity of MgO nanoparticles dispersed in the tryptic soy broth inoculated with overnight cultures of *E. coli* O157:H7 or *Salmonella stanley* has been demonstrated [22]. The toxicity of MgO nanoparticles toward *Escherichia coli* was shown for nanoparticles distributed in Luria–Bertani broth with dispersed bacterial cells [23]. A significant bactericidal effect against *Staphylococcus aureus* was achieved using nanostructured MgO layers, created on Mg substrates using either the anodization of a magnesium substrate or electrophoretic deposition [24]. The films based on acrylic latex comprising embedded MgO nanoparticles were fabricated as an environmentally friendly bactericidal coating [25]. The antibacterial efficiency of such coatings was estimated against a set of bacterial strains, such as *Staphylococcus aureus*, *Escherichia coli*, *Pseudomonas aeruginosa*, *Enterococcus faecalis*, and *Klebsiella pneumoniae*. It was also found that the additives of powdered nanostructured MgO to the latex enabled maintaining a high antibacterial efficiency, which became more pronounced with the increased content of nanoparticles.

A homogenous layer formed through spherical MgF_2_ nanoparticles deposited onto both the inside and outside walls of the catheter was used to protect it against bacterial colonization [19]. These catheters were investigated for their ability to restrict bacterial biofilm formation. It was detected that such catheters can be effective at preventing the bacterial colonization of catheters by *Escherichia coli* and *Staphylococcus aureus* for 1 week, thus indicating long-lasting self-sterilizing properties.

Typically, the analysis of the antibacterial mechanisms induced by magnesium or magnesium-containing nanoparticles in the abovementioned studies [20,21,22,23] revealed the distortion and damage of the bacterial cell’s membrane, resulting in a leakage of intracellular contents and eventually the death of bacterial cells. Furthermore, the role of alkalization of the nanoparticle dispersions, taking place due to magnesium corrosion, was repeatedly discussed as an important mechanism of bactericidal efficiency [20,28,29,30,31].

The excess concentration of Mg ions in the dispersion medium is considered to be another source of the toxic effects of dispersed nanoparticles [30]. To separate the bactericidal impact of Mg^2+^ ions from the high pH effects, the behavior of cultured bacteria *S. epidermidis* and *E. coli* in the dispersions with different concentrations of Mg ions but at a constant pH value (pH = 8.0 ± 0.1) was studied [30]. It was shown that the increase in the concentration of Mg^2+^ is accompanied by a stronger antibacterial effect. The observed phenomena were explained by the large osmotic stress damaging the cell’s wall, yielded by the higher concentration of Mg ions [32].

The next mechanism by which magnesium-containing nanoparticles destroy bacterial membrane structure and functioning is often attributed to the production of reactive oxygen species and its consequences for the bacterial-containing systems [26,33,34,35], although several studies indicate robust toxicity of MgO nanoparticles against bacterial cells in the absence of reactive oxygen species, with no indication of oxidative stress or lipid peroxidation [23,36]. Thus, it may be concluded that the antibacterial action of Mg is based on a multifaceted mechanism.

The production of the nanoparticles for antibacterial purposes, operation with them, uniform distribution in the considered systems, and prevention of their aggregation in a dispersed medium in many cases are not simple tasks. Moreover, efforts are needed to inhibit the removal of the nanoparticles from the touch surfaces. This is why the development of methods of touch surfaces fabrication with tightly fixed nanoparticles and nanotexture elements, which are characterized by bactericidal activity, is highly desirable for the mitigation of hospital-borne pathogenic bacterial growth.

Metal surfaces with a hierarchical roughness conventionally contain nanoparticles or nanotexture elements as characteristic features of the surface. Therefore, the mechanisms of the bactericidal effect of such surfaces should be like those mentioned above and characteristic of the nanoparticles. Furthermore, the additional damaging effect of physico-mechanical forces exerted by the rough surface on the bacterial cell membrane [37,38] significantly contributes to the antibacterial activity. Finally, for non-wetting hierarchical surfaces, two additional mechanisms of antibacterial action come into play, namely the drastically reduced contact area between the substrate and the bacterial dispersion and the decreased adhesion force between the cell and the superhydrophobic surface. The latter inhibits the primary adhesion of the bacterial cells, causing a reduced cell deposition to the superhydrophobic surface and thus reducing bacterial contamination [5,38,39,40].

In this study, we developed a method for the fabrication of superhydrophobic and superhydrophilic magnesium-based surfaces with hierarchical roughness when the surface microrelief is evenly decorated by MgO nanoparticles. The behavior of such surfaces in contact with dispersions of bacteria cells *Pseudomonas aeruginosa* and *Klebsiella pneumoniae* in phosphate-buffered saline (PBS) will be analyzed. Based on the presented data, we will discuss the corrosion and antibacterial activity of the fabricated super wettable surfaces.

## 2. Materials and Methods

### 2.1. Sample Preparation

**Figure 1:**
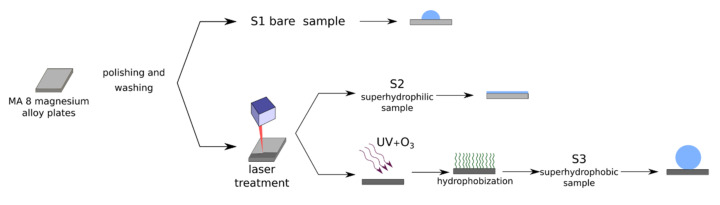
The fabrication procedure for the samples used in this study.

In this paper, three types of samples of MA8 magnesium alloys were studied, including polished metal sheet plates, as well as samples of the same alloys, which were treated to obtain a superhydrophilic or superhydrophobic state of their surface. According to the supplier’s specification (LLC Auremo, St. Petersburg, Russia), the chemical composition of the alloy used was (in weight.%) Mn 1.65, Ce 0.25, Al 0.1, Fe 0.05, Si 0.05, Cu 0.05, Zn 0.04, Ni 0.007, Be 0.002, and Mg balance. For experimental studies of the antibacterial activity, samples with a size of 40 × 40 × 2 mm^3^ were used. For all of the other experiments, areas of 20 × 20 mm^2^ at one end of the sample with a size of 20 × 80 × 2 mm^3^ were subjected to treatment and resulted in either a superhydrophilic or superhydrophobic state. For all of the experiments and samples, the ratio of the sample apparent surface, immersed in dispersion, to the volume of dispersion was kept constant and equal to 0.8 cm^2^/mL. The treatment was applied for both sides of all samples. Before treatment, the samples were ground and polished with a set of SiC abrasive papers, then ultrasonically washed in deionized water and air-dried. To prepare the samples with superwettability, we used nanosecond laser texturing [41]. The sketch of the fabrication procedure for samples used in this study is shown in Figure 1.

To perform the laser processing of the samples, we used an Argent-M laser system (LLC “LTC”, St. Petersburg, Russia) with an IR ytterbium fiber laser and nominal power of 20 W and a RAYLASE MS10 2-axis laser beam deflection unit (RAYLASE GmbH, Wessling, Germany). The laser beam wavelength was 1.064 µm with a beam waist of 40 µm. All of the samples were textured by a single-pass parallel line pattern with a line density of 400 mm^−1^, linear scanning rate of 100 mm/s, pulse duration of 4 ns, repetition rate of 1000 kHz, and a fluence of 1.5 J/cm^2^. The complete spread of water droplets over the surface just after the laser processing indicated the surface superhydrophilicity. To switch the wettability from the superhydrophilic to the superhydrophobic state, the superhydrophilic sample was subjected to UV and ozone treatment for 60 min, followed by the chemical vapor deposition of a fluorosilane CF_3_(CF_2_)_7_CH_2_O(CH_2_)_3_Si(OCH_3_)_3_ as a hydrophobic agent. The chemisorption stage of hydrophobization was performed inside the sealed cell at a temperature of 105 °C.

### 2.2. Preparation of Bacterial Dispersions

For the investigations described in this paper, we used two pathogenic strains, obtained from the State Collection of Pathogenic Microorganisms and Cell Cultures (GKPM-Obolensk, Obolensk, Russia): *Klebsiella pneumoniae B-811* (*K. pneumoniae*, strain registration number B-7707) and *Pseudomonas aeruginosa B-3086* (*P. aeruginosa*, registration number B-8050).

To elucidate the interaction of super wettable (superhydrophilic or superhydrophobic) metallic substrates with bacterial cells without disturbing the influence of protein nutrients [42], we used the dispersions of the bacterial cells in phosphate buffered saline (PBS) with a pH of 7.4 (VWR Life Science AMRESCO, Radnor, PA, USA).

To fabricate the desired bacterial dispersion, 1 mL of freshly prepared culture was centrifuged at 5000 rpm for 5 min. The culture was obtained after 18 h of incubation at 37 °C and was prepared on Luria–Bertani broth (Himedia Laboratories Pvt Ltd., Mumbai, India) with an opacity standard corresponding to 10^9^ colony-forming units (CFU) per mL. The sedimented bacterial pellet was re-suspended three times in 1 mL of PBS and was centrifugally washed at 5000 rpm for 10 min. The desired titer for bacterial contamination (10^7^ CFU/mL) was obtained through sequential ten-fold dilutions with PBS.

### 2.3. Characterization of Bactericidal Properties of the Substrate

To evaluate the antibacterial activity of the superhydrophobic, superhydrophilic, and reference magnesium alloy substrates with respect to each bacterial strain studied, test plates were immersed in separate sterile cups, and 40 mL of bacterial suspension with an initial titer around 10^7^ CFU/mL was poured into each cup. For the control experiments, cups with the same bacterial culture, but without metal plates, were used for both *K. pneumoniae* and *P. aeruginosa*. After a predefined time of contact between each metal plate with the corresponding bacterial suspension, two assays with volumes of 0.1 mL and 1 mL were taken from each cup. The first assay (0.1 mL) was evenly distributed over the surface of Mueller-Hinton agar (Himedia Laboratories Pvt Ltd., Mumbai, India) in Petri dishes. After incubation at 37 °C for 24 h, the bacteria titer was determined. To analyze the effect of the contact of the bacterial dispersion with the bare alloy, superhydrophilic sample, and superhydrophobic sample for each time of contact, the obtained titer was normalized to the initial titer in the dispersion. The results presented below were calculated as the average logarithms of the bacterial titer over two sets of the experiment for each sample and each time duration for the contact of each metal plate with the bacterial suspension. If the results were significantly different between the two sets, additional experiments were performed to obtain a statistically reliable data point.

The second assay (1 mL) was used for the determination of the magnesium egress into the dispersion medium by mass spectroscopy analysis, as described below in Section 2.4. For this, the assay was poured into an Eppendorf tube and centrifuged at 14,000 rpm for 10 min. The liquid phase after centrifugation was used to determine the Mg^2+^ content in PBS.

To check the viability of the bacterial cells adhered to the surface of the samples during 48 h of immersion, the protocol described in detail in [38] was used. Briefly, the samples withdrawn from the bacterial dispersion were rinsed with a sterile physiological solution to remove non-attached cells and then vortexed in 5 mL of physiological solution for 10 min at 150 rpm; then, a 0.1 mL aliquot of the resulting dispersion was applied onto a Petri dish containing sterile growth medium (Mueller-Hinton agar) and conditioned for 24 h at 37 °C. The number of colony-forming units was determined after that.

### 2.4. Determination of Magnesium Concentration in the Dispersion Medium

Mass spectroscopy experiments were applied to determine the magnesium and phosphorus ion concentrations in the dispersion medium at different times of contact of magnesium alloy substrates with bacterial dispersion and with PBS. We used an inductively coupled plasma mass spectrometer (ICP-MS) Agilent 7500CE instrument (Agilent Technologies, Santa Clara, CA, USA) featuring the “ICP-MS-Top” program for data acquisition. The instrument operating regime was adjusted as discussed earlier [42].

High-purity-grade concentrated nitric acid (65 w%, Sigma-Aldrich, Burlington, VT, USA) was added to each sample to obtain the 0.01 M solution. Then, the digestion of samples was carried out at 60 °C for 40 min. After the above steps, the resulting solution was injected directly into the instrument. Laboratory blank samples were also analyzed for the same digestion method. The average magnesium and phosphorus concentrations in the blank samples were then subtracted from the measured concentration in each digest to give the final reported concentration. It was shown that the background concentration of the magnesium was less than 3 μg/L. Each sample was analyzed twice to assess the possible drift effects.

### 2.5. Characterization of Dispersion Medium pH upon Contact with Metal Plates

To trace the variation in pH upon contact of our substrates with either PBS or the bacterial dispersion, which contained several corrosion-active entities, test plates were immersed in separate sterile cups in the liquid phase, with a ratio of the apparent sample surface to the liquid volume equal to 0.8 cm^2^/mL. After predefined times of contact of each metal plate with the liquid medium, the pH was measured in each cup using microelectrode ESK-10614 (LLC Measuring Technology, Moscow, Russia).

### 2.6. Characterization of Surface Wettability

Wettability of coatings before and after immersion into bacterial suspensions were characterized by measuring the contact angles for the bare and superhydrophilic samples. For the superhydrophobic samples, both the contact and roll-off angles were measured. Prior to performing the wettability measurements for the samples after contact with the bacterial dispersions, the samples were decontaminated in an oven at 180 °C for 30 min. Digital image processing of sessile droplets and Laplace fit optimization for determining the droplet shape parameters were used to measure the advancing contact angles [43,44]. Roll-off angles for sessile droplets were defined upon substrate tilting until the droplet started to move. Both the contact angles and roll-off angles were measured for 15 μL droplets at least at 10 different surface locations for each sample.

### 2.7. Surface Morphology and Composition Characterization

The morphology of the bare, superhydrophobic, and superhydrophilic surfaces of magnesium alloy before and after contact with bacterial dispersion and the structures formed on these surfaces as a result of the interaction of the surface with bacterial cells and buffer solution were investigated by field-emission scanning electron microscopy (FE-SEM) and energy-dispersive X-ray spectroscopy (EDX) on a FIB-SEM Nvision 40 workstation (Zeiss, Oberkochen, Germany) equipped with X-MAX energy-dispersive detector (Oxford Instruments, High Wycombe, UK). The FE-SEM images were recorded in secondary electron (SE) detection mode at accelerating voltages of 2−5 kV. EDX microanalysis was performed at 10 kV accelerating voltage.

### 2.8. Data Analysis

Statistical data processing was performed with built-in statistical tools of Microsoft Excel 2010 and Statistica 8.0. One-way analysis of variance was performed to determine significant group differences, and means were considered statistically significant if *p* < 0.05.

## 3. Results and Discussion

### 3.1. Wettability

The procedure used to prepare the superhydrophilic and superhydrophobic samples as listed in Section 2.1 was chosen based on the highest corrosion resistance of the samples in a superhydrophobic state, which could be attained through the selection of numerous laser treatment parameters. Such an analysis was performed in our recent study and is described there in detail [41]. The samples used for the studies of the antibacterial activity were first characterized according to their wettability. It is worth noting that freshly prepared superhydrophobic and superhydrophilic samples had a light gray color and were evenly colorized. The water contact angles measured for the samples before and after 48 h of contact with PBS are presented in Table 1.

As can be seen from the presented data, the bare MA8 sample was hydrophilic. Laser texturing at the chosen parameters of the laser treatment resulted in establishing the superhydrophilic state with complete water droplet imbibition inside the texture. After the deposition of fluorooxysilane from the vapor onto the superhydrophilic surface, the samples demonstrated a superhydrophobic state with very high water contact angles and low roll-off angles. As discussed below, the designed superhydrophobic state persisted after 48 h of contact with a very corrosive environment such as PBS. The comparison of the wettability for three types of substrates (bare, superhydrophilic, and superhydrophobic), freshly prepared and after 48 h of immersion into PBS, indicated a notable difference. Thus, the bare plate after prolonged contact with PBS became covered by dark gray corrosion products and demonstrated nonuniform wetting along the surface with an average water contact angle of around 30°. After subsequent heat treatment in an oven at 180 °C for 30 min, the water contact angle increased to 49°, as shown in Table 1. At the same time, the wide variation of the contact angles along the surface persisted.

The superhydrophilic sample after contact with PBS changed its coloration from light gray to white due to covering by white deposits. Water droplets deposited onto the superhydrophilic surface air-dried for 30 min after immersion in PBS demonstrated complete spreading. The high-temperature treatment of the sample led to some deterioration of a superhydrophilic state. After heat treatment, the superhydrophilic sample showed hemi-wicking of water droplets with a contact angle of 10.0 ± 5.0°.

In contrast, the superhydrophobic sample showed the appearance of separate areas of white deposits, with a very poor adhesion of these deposits to the underlying superhydrophobic layer. The gray areas on the superhydrophobic sample represented the regions with a moderate deterioration of the contact angle from the value around 171° to 163 ± 1° and a notable increase in roll-off angles. White areas were characterized by a hemi-wicking wetting regime with a water contact angle of 20 ± 10°. During the contact angle measurement, the white deposits were easily transferred from the sample surface to the water droplet through capillary forces. Sample heating at 180 °C for 30 min returned the sample to its original wettability state with a very high contact angle and low roll-off angle. As the observed variation of the wetting state can be related to variation in the sample morphology due to the chemical interaction of the magnesium alloy substrate with PBS, we studied the morphology and composition of our samples after contact with the phosphate buffered saline.

The immersion of samples into both bacterial dispersions led to the variation of the contact angles along the samples, being notably higher than in the case of PBS, while the average values for each sample were nearly the same for both bacterial strains.

### 3.2. Surface Morphology of Samples, Subjected to Immersion in PBS 

**Figure 2:**
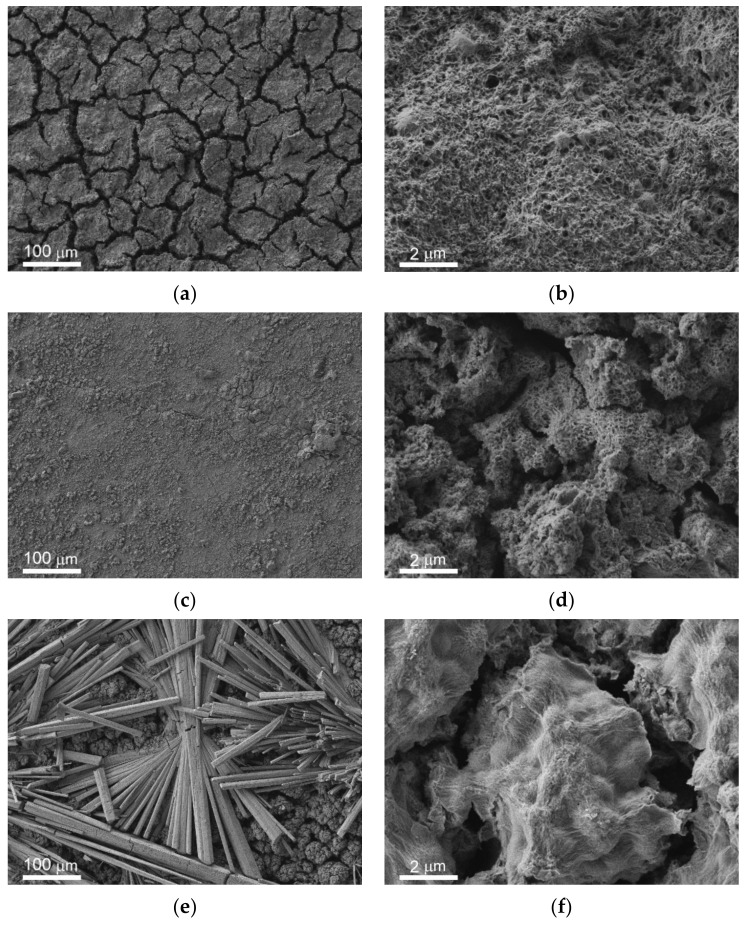

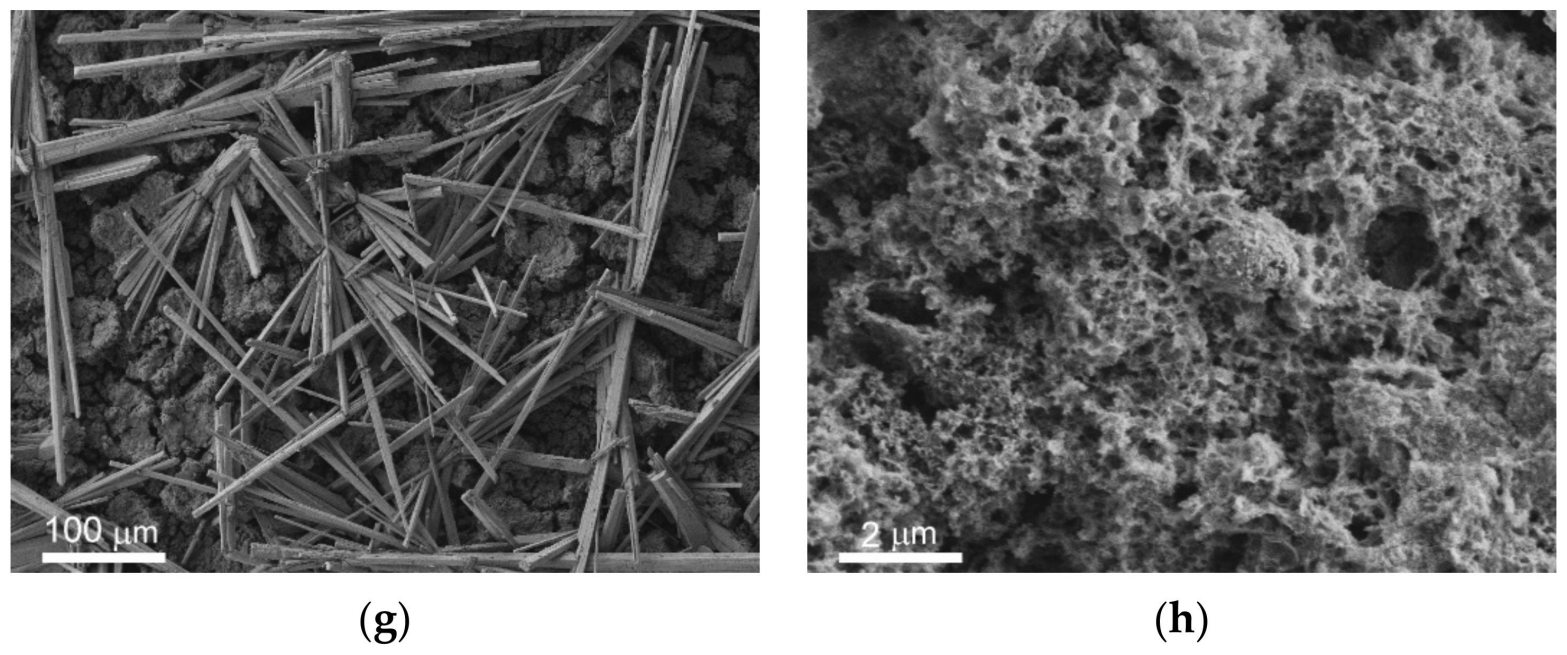
SEM images with different magnifications illustrating the morphology of magnesium alloy samples with different wettabilities before (**a**,**b**) and after (**c**–**h**) immersion for 48 h into a phosphate buffer solution (PBS): (**a**,**b**) laser-textured superhydrophilic or superhydrophobic samples; (**c**,**d**) polished bare MA8 sample; (**e**,**f**) superhydrophilic sample; (**g**,**h**) superhydrophobic sample. The images are arranged as follows: left column (**a**,**c**,**e**,**g**) presents survey images with a scale bar of 100 μm; right column images (**b**,**d**,**f**,**h**) show the details of the surface structure at a larger magnification with a scale bar of 2 μm.

The overview SEM images of the bare, superhydrophilic, and superhydrophobic samples after 48 h of immersion in PBS are shown in Figure 2c,e,g, respectively. For the comparison, the morphology of the laser-textured surface (which was similar for both superhydrophilic and superhydrophobic surfaces) before immersion is given in Figure 2a,b. The well-developed and rough surface of the bare sample after immersion (Figure 2c) is quite different from the initial polished surface. Such morphology changes are evidently related to the electrochemical processes at the interface during the contact of the bare MA8 alloy with corrosive PBS. EDS analysis and FT-IR spectroscopy indicate that the surface is enriched with magnesium oxide and hydroxide, as well as with magnesium phosphates containing a small amount of sodium and potassium. The formation of the composite micro and nanotexture (Figure 2c,d) on top of the bare alloy surface as a result of chemical interaction with PBS allows for explaining the decrease in the contact angle value due to roughness increase.

The morphology of the superhydrophilic sample shown in Figure 2e allows concluding that the white deposits visible with the naked eye are associated with the huge number of bundles of microrods formed on the surface during contact with PBS. These rods are constituted by hydrophosphate of magnesium and mixed hydrophosphates of magnesium with sodium and potassium. Besides, the rods-free part of the substrate is also covered by the layer, which was newly formed. The formation of this additional layer that covered the superhydrophilic surface is seen in Figure 2f. Since the newly formed layer is also characterized by the multimodal texture, the complete wetting regime was kept after the contact of the superhydrophilic surface with PBS. The presence of microrods on top of such a surface just after withdrawal from PBS contributed to the preservation of the complete wettability. However, a heat treatment, which stimulated the transition of hydrated forms of surface compounds to water-depleted ones, caused some deterioration of the hydrophilicity with hemi-wicking of the deposited droplet (Table 1).

Images of the superhydrophobic sample after contact with PBS indicate the formation of a composite layer covering the surface texture for gray areas and many bundles of microrods on top of the covering layer on white areas (Figure 2g,h). However, the number of rods and their sizes for the superhydrophilic surface were significantly higher in comparison with the superhydrophobic one. At the same time, the morphology of the newly formed layer satisfied the condition of multimodality, and the transfer of hydrophobic agent from the pores of the initial superhydrophobic surface to the newly formed layer, made it superhydrophobic as well. This tendency was partially counterbalanced by the formation of the white patches, constituted by hydrophilic rods, which made the surface less water-repelling and caused an increase in roll-off angles (Table 1). Enhanced diffusion of the hydrophobic agent from the pores onto the very top layers during heat treatment at high temperatures led to the enrichment of the surface of newly formed textures with the hydrophobic agent. Besides, the rather weak adhesion of rods to the surface resulted in their spontaneous partial removal. Therefore, during heat treatment, the above processes caused an increase in contact angle and a decrease in roll-off angle.

### 3.3. Anti-Bacterial Activity of Magnesium Alloy Samples

One of the key goals of this work was to study the bactericidal activity of MA8 alloy samples characterized by extreme wettability, or in other words, superhydrophobic and superhydrophilic samples, with respect to the pathogenic bacterial cells of *P. aeruginosa* and *K. pneumoniae*. It was shown in our recent studies [40,42] that the type of the dispersion medium significantly affects the antibacterial activity of the metallic surfaces immersed into the bacterial dispersion because of its impact on the corrosion activity of such surfaces. Microorganisms and proteins are able to change the electrochemical conditions at the metal/bacterial dispersion interface by either biofilm formation or by adsorption of proteins from a nutrient dispersion medium. In particular, the protein components of Meat-Peptone broth affected the bactericidal properties of the metal substrates through the intensification of the corrosion processes. The corrosion rate in the nutrient medium free from bacterial cells was found to be higher than in the 0.5 M NaCl solutions [42].

To reveal the mechanisms of the antibacterial properties of our substrates in more detail, in this work we used dispersions of cells purified from the protein components of the nutrient medium using PBS. In this set of experiments, we monitored the temporal evolution of the titer of living cells in glass cups, either in contact with a metal substrate or not.

For comparison, the normalized titer of the suspension with both cultures, *K. pneumoniae* and *P. aeruginosa* stored at the same conditions, but in the absence of metal, was measured simultaneously and served as a control. The cups were stored at room temperature and after a predefined time of contact between each metal plate with the corresponding bacterial suspension, an assay with a volume of 0.1 mL was taken from each cup to determine the actual titer of the living cells, as described above. To quantify the bactericidal effect, for each time of contact, the obtained actual titer was normalized to the initial titer in the dispersion (10^7^ CFU/mL). Two sets of experiments for each sample and each time duration for the contact of a metal plate with the bacterial suspension were performed to obtain a statistically reliable titer.

**Figure 3:**
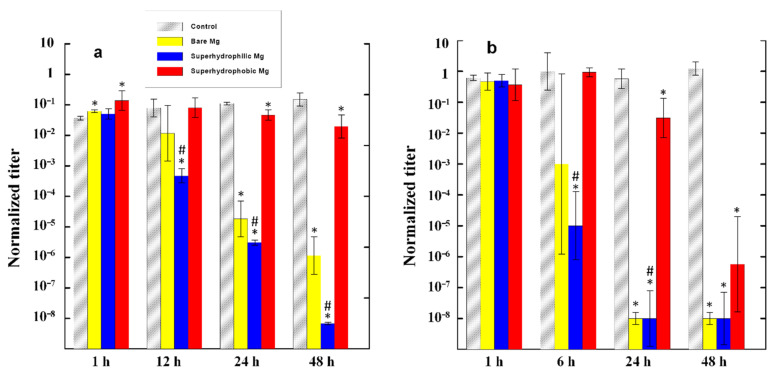
Evolution of the titer of *P. aeruginosa* (**a**) and *K. pneumoniae* (**b**) in cups with bacterial suspension with a time of contact between the suspension and MA8 plates with different wettability: polished MA8 alloy (yellow posts), superhydrophilic (blue posts), and superhydrophobic (red posts) coatings. The reference gray posts represent the corresponding variation of titer in the control bacterial suspension. Error bars represent the standard deviations of the logarithm of the bacterial titer; * *p* < 0.05 compared with the blank control; ^#^ *p* < 0.05 indicates a significant difference between the superhydrophobic and the superhydrophilic substrates.

The evolution of the number of survived cells for both bacterial strains is shown in Figure 3. The analysis of the evolution of the bacterial titer in the control bacterial dispersions for both bacterial strains indicated that the number of living cells remained nearly the same (*K. pneumoniae*) or weakly increased (*P. aeruginosa*) with time during 48 h of monitoring. Thus, PBS can be considered as a friendly medium for bacterial cell growth.

The data presented in Figure 3 convincingly show that among the three studied metallic samples, the maximum antibacterial activity was characteristic of the superhydrophilic plates. For example, when such plates were immersed in the *P. aeruginosa* dispersion, after 24 h of contact, the decontaminated dispersion contained six orders of magnitude less living bacterial cells than the control dispersion that did not contact the magnesium alloy, and five orders of magnitude less than the initial dispersion. Considering that the initial titer of the bacterial cells used for the experiment was of the order of 10^7^ CFU/mL, the concentration of viable pathogenic bacteria in dispersions after 24 h of contact with the superhydrophilic plates can be considered as relatively benign. The bactericidal efficiency of a polished MA8 plate was lower than that of a superhydrophilic plate. Nevertheless, after 24 h, the dispersions of *K. pneumoniae* in contact with superhydrophilic and polished MA8 plates had nearly the same level of bacterial contamination.

In the first hours of contact with the dispersion, the superhydrophobic plate did not exhibit a bactericidal activity with respect to the bacterial cells dispersed in a liquid medium, and only began to noticeably reduce the titer of bacterial cells after nearly 24 h of contact. Although after 48 h the titer of bacteria in the dispersion with the immersed superhydrophobic plate was significantly reduced, both with respect to the control dispersion and relative to the initial titer, the bactericidal efficacy of the superhydrophobic plate MA8 remained significantly lower than that of the superhydrophilic one, especially in the case of the dispersion of *K. pneumoniae*. Except for the very short times of contact (1 h) and 48 h in the dispersion of *K. pneumoniae*, the differences between the mean values of bacterial titers for the superhydrophilic and superhydrophobic samples were statistically significant with *p* < 0.05. To reveal the mechanism of the bactericidal action of MA8 plates with different wettability, we performed a series of additional experiments discussed below.

### 3.4. Variation in pH for PBS Solution Contacted with Magnesium Samples Having Different Wettability

As discussed above, the contact of the magnesium alloy with a corrosive medium is accompanied by metal dissolution, hydrogen release, and the variation in pH of the liquid medium. In turn, pH evolution modifies the interaction of metal surfaces with the components of the liquid medium and with bacterial cells, and, as such, should affect the antibacterial activity of the magnesium alloy substrates with different wettability. The contact of magnesium or its alloys with an aqueous solution is accompanied by the chemical reaction

Mg + 2H_2_O → Mg^2+^ + 2OH^−^ + H_2_ ↑, (1)

which causes alkalization of the liquid and hydrogen release.

The increase in alkalinity caused by the corrosion degradation process is considered as one of the main mechanisms of bactericidal activity of magnesium-based materials [20,28,29,30,31,45].

It has been shown that cell lysis and loss of vital intracellular contents at a high pH is related to two mechanisms [45], namely disruption of the cytoplasmic membrane followed by the leakage of the internal contents and phase separation of the cytoplasm. Besides, the alkalization of the liquid medium enhances the chemical stability of reactive $O_{2}^{-}$ and inhibits the formation of hydroperoxyl radical ${\mathrm{HO}}_{2}^{•}$.

**Figure 4:**
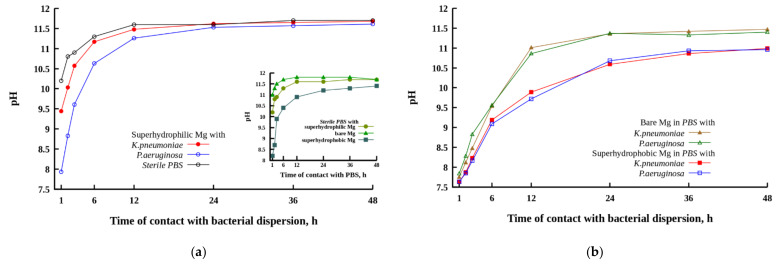
Variation of pH with a time of contact between bacterial dispersions and MA8 alloy sample with the superhydrophilic coating (**a**), and the superhydrophobic coating and bare MA8 alloy (**b**). Inset shows the time evolution of pH in sterile PBS with the immersed superhydrophilic, superhydrophobic, or bare sample. In the legend, “PBS” denotes phosphate buffer solution without bacteria, and the bacteria names denote the dispersion of the corresponding bacterial strain in PBS. Lines are only to guide eyes.

The evolution of pH in cups containing MA8 plates with different wettability immersed in PBS and bacterial dispersions is illustrated in Figure 4. For such experiments, the ratio of the plate apparent surface area to PBS volume was chosen to be the same (0.8 cm^2^/mL) as in experiments in order to study the bactericidal action of magnesium plates with respect to bacterial cells dispersed in PBS. The inset in Figure 4 shows the variation of pH in sterile PBS with the time of contact with different MA8 samples. The slowdown of the alkalization of the liquid nutrient medium in the presence of bacteria is clearly presented in Figure 4a.

During the first hour of contact, the most intense pH increase in sterile PBS was detected for the polished magnesium alloy plate (inset in Figure 4). After 6 h, the plateau value with pH~11.8 was reached. The low solubility magnesium compound deposits appeared in the cup during 1 h. For the superhydrophilic plate in contact with PBS, the plateau value with a pH = 11.6 was reached after 12 h. This behavior is evidently related to the presence of the top micro and nanotextured MgO layer, formed during laser treatment [41]. Although this layer is not dense and continuous, it enhances the barrier properties of the metal plate and provides some limited protection against magnesium dissolution in PBS in conditions of a more developed surface area compared with the polished sample. The highest protective properties against dissolution in a highly corrosive medium were revealed by the superhydrophobic plate. The deposits of low-soluble magnesium compounds appeared in the cup after 12 h of exposure to the superhydrophobic sample in PBS.

In the presence of bacterial cells in a PBS dispersion medium, the corrosion reaction on the surface of MA8 with different wettability was somewhat inhibited, leading to a slowdown of pH growth with the time of sample’s contact with the bacterial dispersion (Figure 4a,b). The inhibition of microbiologically induced corrosion was less notable for the superhydrophilic magnesium sample in contact with the *K. pneumoniae* dispersion. Although at a short time of contact, the alkalinity in the bacterial dispersion was lower than in sterile PBS, the pH value established in liquid after 48 h of the superhydrophilic sample immersion was nearly the same as for the sample in the sterile solution. At the earlier stage of contact of the metal samples with a bacterial medium, the highest difference in pH versus bacteria-free medium was detected for the bare sample, while at prolonged contact, the highest protective behavior in bacterial dispersions was shown by the superhydrophobic sample. It is interesting to note here that for the superhydrophilic and bare samples, the corrosiveness of bacterial dispersion of *K. pneumoniae* was higher than that for the dispersion of *P. aeruginosa*.

Now let us consider the evolution of pH in all types of liquid media including the sterile PBS and both bacterial dispersions in contact with the superhydrophobic MA8 sample. The mechanisms of corrosion inhibition by the superhydrophobic surfaces were discussed recently [46]. Four mechanisms should be mentioned here. These include water-repelling properties leading to a decrease in liquid/surface contact area and the development of a high negative capillary pressure preventing the imbibition of the aggressive liquid into the oxide layer. The enhanced barrier properties of both MgO and the self-organized layer of the hydrophobic agent reduce the transfer of corrosive entities to the metal surface. The chemisorbed molecules of the hydrophobic agent block chemically active sites on the surface, thus hindering the adsorption of corrosive ions and inhibiting the corrosion process. Finally, negative charging of a superhydrophobic surface at neutral or alkaline conditions resulted in the repulsion of corrosive anions from the surface. Due to the above factors, the enrichment of liquid media by magnesium, and hence, by hydroxide ions in the cups with the superhydrophobic samples, was slowed down (Figure 4b), and the final pH values were the lowest, although still significantly exceeded the pH ≈ 9 level, which was discussed in the literature as causing bacterial death [20,30].

An additional point that should be discussed here is related to the level of pH achieved in our experiments after prolonged contact between the MA8 samples with different wettability and bacterial dispersions in PBS. The state of magnesium hydroxide formed in reaction (1) depended on the pH of the liquid medium, and for pH > 10.5 it typically precipitates due to low solubility. Our data indicate the onset of the precipitation at a notably higher pH. The reason for such behavior may be related to the high concentration of chloride ions in the PBS solution. When the Cl^−^ concentration reaches the level of 150 mmol/L, exchange reactions result in the formation of highly soluble potassium and sodium hydroxides, and magnesium chloride. Besides, magnesium hydroxide is not the only degradation product that is expected in the PBS solution. The presence of phosphate ions causes the precipitation of magnesium phosphate and hydrophosphates, as well as mixed magnesium/sodium/potassium hydrophosphates. Keeping in mind the possible impact of magnesium ions’ concentration on the viability of bacterial cells in dispersions, it was reasonable to measure the concentration of Mg^2+^ versus time of immersion of MA8 plates with different wettability in PBS.

### 3.5. Variation in Mg^2+^ Concentration during Contact of Magnesium Samples Having Different Wettability with PBS or Bacterial Dispersions

The multicomponent composition of the considered systems containing the magnesium alloy plate and PBS or bacterial dispersion in PBS, with the products of chemical interaction between the magnesium and the liquid phase, did not allow for unambiguously relating the pH of the liquid medium with the concentration of magnesium ions. At the same time, the ion concentration was considered as one of the determining factors for the bactericidal action of metals for bacterial cells, both planktonic and deposited onto the substrate [20,30,31,32]. For example, the bactericidal effects of magnesium ions and the solution pH were differentiated in [32], and it was shown that a higher concentration of magnesium ions resulted in a more intense bactericidal effect, which the authors had related to the larger osmotic stresses exerted on the cells.

The evolution of the concentration of magnesium and phosphorus obtained in liquid PBS without bacteria, monitored as described in Section 2.4, is discussed in the Supplementary Materials and presented in Figure S1 for three cups containing polished bare, superhydrophilic, or superhydrophobic MA8 samples.

The data and the analysis presented in the Supplementary Materials indicate that the concentration of magnesium ions in PBS contacted with the samples of different wettability during the whole immersion time remained notably lower than those discussed in the literature as toxic ones [28,32].

A similar analysis of the evolution of Mg^2+^ ions concentration in a liquid medium in contact with the superhydrophobic metal plate was also performed for bacterial dispersions in PBS. In this series of experiments, the bacterial dispersion after a predefined time of contact with the metal sample was centrifuged at 14,000 rpm for 10 min, the liquid fraction was separated from bacterial cells and sediments, and was subjected to UV treatment for 1 h to ensure bacterial decontamination. Then, the Mg^2+^ ions concentration was measured using mass spectroscopy.

**Figure 5:**
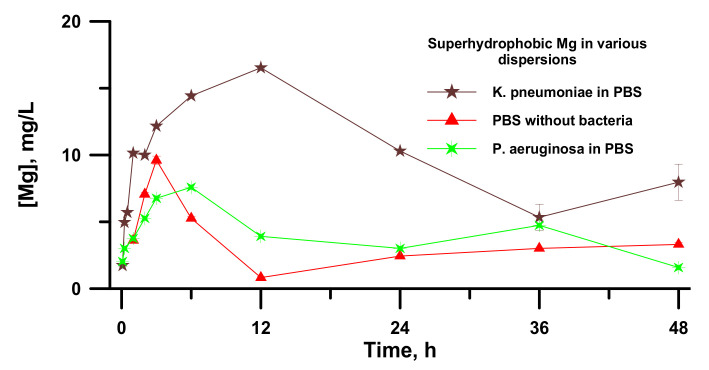
Time evolution of the magnesium content in PBS-based bacterial dispersions contacted with a superhydrophobic MA8 sample. Error bars represent the standard deviations.

For the superhydrophobic magnesium samples contacted to either bacteria free PBS or dispersions of *P. aeruginosa* and *K. pneumoniae* in phosphate-buffered saline, the variation of concentration with an increase in time of immersion of a sample is presented in Figure 5.

As discussed in the literature, when considering the heterogeneous ice nucleation [47,48], the heterogeneous wetting regimes of a superhydrophobic surface by an aqueous liquid medium and high contact angle formed by aqueous droplet with such a surface results in the enhanced barrier for the nucleation of a new phase, such as magnesium phosphates. A high nucleation barrier, in turn, causes the inhibition of the new phase formation and the increased value of Mg^2+^ concentration, which is necessary for the beginning of new phase growth. The constancy of the phosphorus concentration (Figure S1a) in line with the increase in the concentration of magnesium (Figure 5) during 6 h of contact between the superhydrophobic plate and PBS conforms to the statement about an increase in the barrier of nucleation of magnesium phosphates from the aqueous phase on top of the superhydrophobic sample. Reaching some critical concentration of magnesium ions leads to the nucleation and growth of magnesium phosphate crystals and a sharp decrease in magnesium concentration in the solution, simultaneously with a decrease in the phosphorus concentration. After 36 h of contact between the PBS and the superhydrophobic sample, the deposition of non-soluble reaction products onto the magnesium surface leads to the diffusion inhibition of magnesium dissolution through a porous passive film formed on its surface. Thus, the combination of different processes results in establishing the stationary state in magnesium dissolution and new magnesium phase formation in bacteria free PBS solutions.

As for the bacterial dispersions, it was found that at a short time of contact, the magnesium concentration increased, while the dispersion remained transparent. However, after reaching a critical concentration, the Mg^2+^ concentration in PBS began to decrease at a simultaneous decrease in phosphorus concentration (not shown in Figure 5), indicating the formation of magnesium phosphates, similar to that in PBS without bacterial cells. After 6 h for the dispersion of *P. aeruginosa* and after 12 h for the dispersion of *K. pneumoniae*, the surface of the superhydrophobic sample became covered with patches of white deposits, and the dispersion medium turned into an opaque state. To better understand the results of the interaction between the bacterial cell dispersions and the samples with different wettability, we studied morphology and elemental composition of our samples after 48 h of immersion in dispersions of *P. aeruginosa* and *K. pneumoniae*.

### 3.6. Morphology and Elemental Composition of Metal Samples Contacted to the Bacterial Dispersions

**Figure 6:**
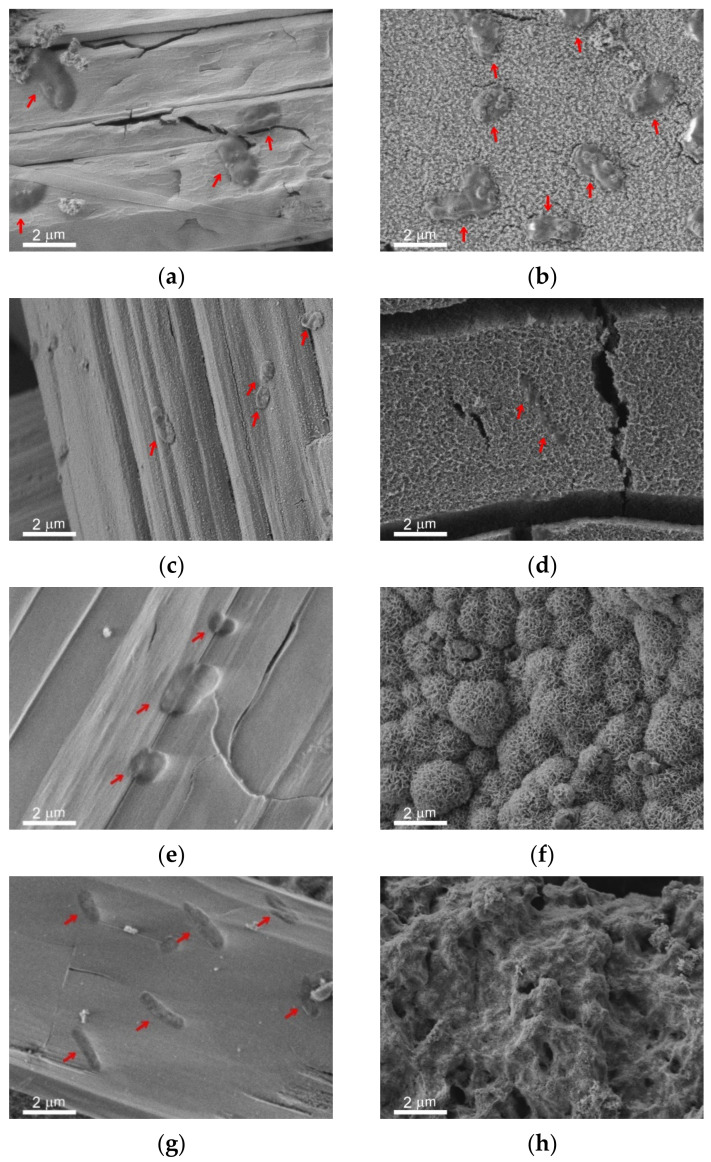

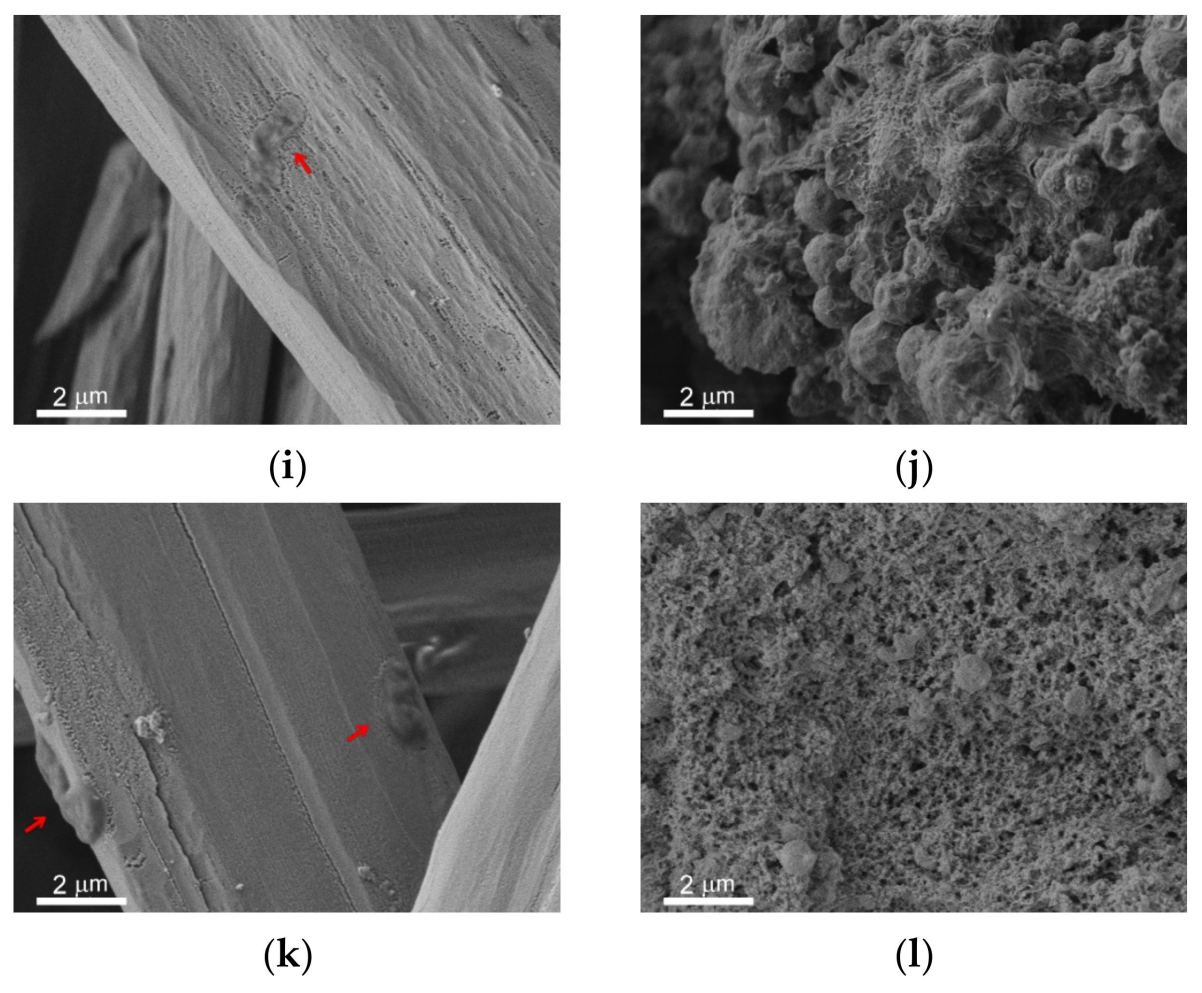
SEM images illustrating the morphology of the magnesium alloy samples with different wettability after immersion for 48 h into the bacterial dispersions in PBS. The images are arranged as follows. The horizontal rows of images correspond to different types of substrates and immersion liquids: (**a**,**b**) polished bare MA8 sample after immersion into the dispersion of *K. pneumoniae*; (**c**,**d**) polished bare MA8 sample after immersion into the dispersion of *P. aeruginosa*; (**e**,**f**) superhydrophilic sample after immersion into the dispersion of *K. pneumoniae*; (**g**,**h**) superhydrophilic sample after immersion into the dispersion of *P. aeruginosa*; (**i**,**j**) superhydrophobic sample after immersion into the dispersion of *K. pneumoniae*; (**k**,**l**) superhydrophobic sample after immersion into the dispersion of *P. aeruginosa*. Left column (**a**,**c**,**e**,**g**,**i**,**k**) presents magnified images of microrods deposited on the covering layer; right column images (**b**,**d**,**f**,**h**,**j**,**l**) show the structure of the layer covering the intrinsic texture of the substrate. Red arrows point bacterial cells deposited onto the rods or covering layers.

The immersion of metal samples with different wettability into the bacterial dispersions in PBS led to the formation of the covering layer and microrods on all surfaces. The survey SEM images of the surfaces of the studied samples are shown in Figure S2 in Supplementary Materials. In Figure 6, the peculiarities of microrods distribution over the different surfaces are presented in the left vertical column (Figure 6a,c,e,g,i,k). The right column (Figure 6b,d,f,h,j,l) elucidates the structure of the covering layer located under the rods on top of the intrinsic substrate texture. The horizontal rows of images correspond to different types of substrates and immersion liquids: first row (images a,b) is polished bare MA8 sample after immersion into the dispersion of *K. pneumoniae*, second row (c,d) is polished bare MA8 sample after immersion into the dispersion of *P. aeruginosa*, third row (e,f) is superhydrophilic sample after immersion into the dispersion of *K. pneumoniae*, fourth row (g,h) is superhydrophilic sample after immersion into the dispersion of *P. aeruginosa*, fifth row (i,j) is superhydrophobic sample after immersion into the dispersion of *K. pneumoniae*, and sixth row (k,l) is superhydrophobic sample after immersion into the dispersion of *P. aeruginosa*.

**Figure 7:**
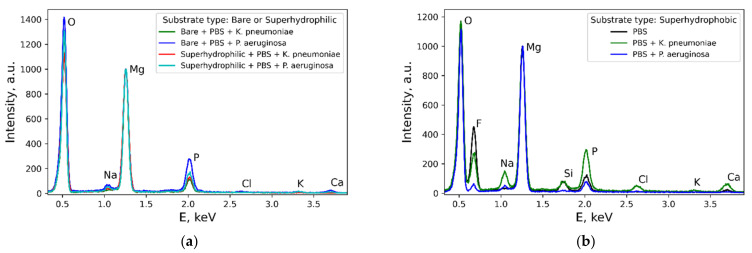
EDS spectra illustrating the elemental composition of the surface of metal samples with different wettability after 48 h of contact with bacterial dispersions: (**a**) Bare and superhydrophilic samples; (**b**) superhydrophobic samples. In the legend, “PBS” denotes a phosphate buffer solution without bacteria, and the bacteria names denote the dispersion of the corresponding bacterial strain in PBS.

The comparison of the sample morphology after contact with bacterial dispersion in PBS and with PBS free from the bacterial cells (Figure 2) indicates a notable difference. Thus, for bare samples, nano and microrods were only formed in the bacterial dispersions. The morphology of the covering layers was shown to be significantly affected by the type of bacterial strains and the sample wettability (Figure 6 b,d,f,h,j,l). Some variation with the wettability was observed for the layer composition (Figure 7). In contrast, the composition of rods formed on different substrates contacted with either of the two considered types of bacterial dispersions was nearly the same.

Now let us briefly discuss the deposition of bacterial cells onto the samples. As can be seen from the images for the bare and superhydrophobic samples, an abundant amount of bacterial cells was deposited onto the rods for both bacterial strains (marked by red arrows in Figure 6a,c,i,k). The morphology of cells deposited onto the rods was notably perturbed, less explicitly for the *K. pneumoniae* cells on the bare sample. As for the bacterial cells adhered to the covering layers, such cells were only found on the surface of the bare samples, and the destruction of such cells (Figure 6b,d; marked by red arrows) can be concluded.

### 3.7. The Role of Various Factors in the Bactericidal Effect of Magnesium Alloy

The use of the significant antibacterial effect of materials based on magnesium and its alloys, which has been repeatedly discussed in the literature [16,17,18,19,20,21,22,23,24,25,26,27], is hampered by the fact that despite numerous studies, there is still no consensus on the mechanisms that determine this effect. In this work, along with the analysis of such mechanisms, we focused on issues that have remained practically unexplored until now. One of the main questions of this kind was the study of the influence of the extreme wettability of material on the effectiveness and mechanisms of its bactericidal action. The results described above were obtained for the polished MA8 alloy, and for laser-treated superhydrophilic and superhydrophobic samples.

Our studies have shown that the interaction of the highly reactive surface of the magnesium alloy with the buffer solution caused the change in both the composition and morphology of the surface layer. Thus, a polished sample showed a decrease in the contact angle due to an increase in roughness and the formation of a distinct texture. The presence of bacterial cells for both studied strains in the liquid medium in contact with the sample further reduced the contact angle. Superhydrophilic and superhydrophobic samples also significantly changed their morphology after exposure to PBS and even more significantly after exposure to bacterial dispersions in PBS. On both types of extreme wettability surfaces, the formed layers and bundles of microrods also contributed to a multimodal texture. Therefore, the superhydrophilic sample retained its superhydrophilic properties even after prolonged contact with bacterial dispersions. As for the superhydrophobic sample, the formed layer and rods were hydrophilic, as they consisted of magnesium and mixed phosphates of magnesium, sodium, and potassium. Therefore, when a superhydrophobic sample was immersed in bacterial dispersions for a long time, and areas with the covering layer and rods increased so much that they covered a significant part of the initially superhydrophobic surface, the sample lost its superhydrophobic properties and passed into a hydrophobic and then a hydrophilic state. In addition, due to the interaction of the superhydrophobic surface with a corrosive bacterial environment, some degradation of the superhydrophobic surface itself occurred with the dissolution of magnesium and the appearance of wetting defects. This could be traced by the evolution of hydrogen and turbidity of the liquid medium. It is interesting to note that if such a sample, which initially had superhydrophobic properties and lost its superhydrophobic properties after contact with bacterial dispersions, was washed with a strong jet of water, then the covering layer and rods were removed, and the surface restored water-repellent properties with a contact angle >150° and water droplets rolling off the surfaces with roll-off angles not exceeding 15°. This behavior allows for concluding that despite the apparent degradation of the wetting properties, the magnesium base of the coatings remains largely intact and the egress of magnesium products into the environment remains limited. The behavior of the water droplets on top of superhydrophobic surfaces contacted with a bacterial dispersion before and after water jet washing is presented in Supplementary Video S1.

A joint analysis of changes in the titer of bacterial cells and pH in PBS in contact with magnesium samples showed that for all magnesium samples studied in this work, the bactericidal effect against both bacterial strains was observed, while the maximum antibacterial activity was manifested under the conditions of the highest pH values of the dispersion medium.

**Figure 8:**
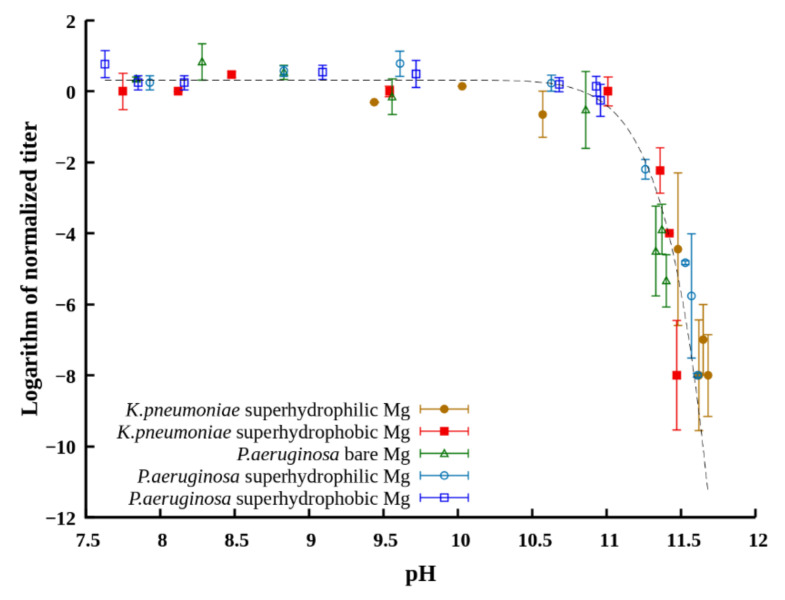
The dependence of the bacterial titer in the bacterial dispersion contacted for different times with the magnesium substrate on the pH of the dispersion medium. In the legend, bacteria names denote the dispersion of the corresponding bacterial strain in PBS. The dashed line bearing a feature of the “master curve” is a least-square power-law fit. Error bars represent the standard deviations.

To substantiate the role of increased pH in the bactericidal effect, we plotted the titer of planktonic bacteria in the dispersion after different times of contact with the tested samples for both studied strains versus the pH value established at these times in the dispersion medium (Figure 8). The sets of data obtained for the samples with different wettability in dispersions of both *K. pneumonia* and *P. aeruginosa* are well described by the same master curve. This allows for discriminating the bacteriostatic and bactericidal range of pH in liquids with immersed magnesium, where pH is determined by induction of corrosion processes on the metal surface. Thus, the value around pH = 11 can be considered as a critical one, designating the boundary between the bacteriostatic and strong bactericidal effect of the surrounding dispersion medium.

Another important mechanism of the anti-bacterial action of magnesium plates immersed into the bacterial medium is related to the collection of bacterial cells by the microrods. As discussed above, these rods formed on the plates as a result of the chemical interaction of magnesium with the components of the nutrient medium. As seen on the images presented in Figure 6a,c,e,g,i,k, a large number of cells was deposited onto the surface of the rods.

The analysis of the cell’s morphology, shown in Figure 6a,b,d,g,i,k (See also the Supplementary Figure S3), indicates the cell degradation and flattening. The more significant effect, presented in Figure 6b,d,g,i and associated with a decrease in cell thickness, is seemingly related to membrane damage, followed by a release of an intracellular liquid. The latter results in cell flattening. To substantiate the death of the cells adhered to the rods, we checked the viability of deposited cells as described in Section 2.3. The absence of colony-forming units on top of the growth medium (Mueller-Hinton agar) after 24 h of exposure at 37 °C confirmed the death of the bacterial cells sorbed by the rods. As the surface of the rods is relatively flat and does not contain sharp-edge texture elements, it is reasonable to relate the death of sessile (deposited) bacterial cells with the high pH value. As was mentioned above, the alkalization of a liquid medium causes the degradation of the cell membrane through the destruction of chemical bonds, phase separation of the cytoplasm, and enhances the chemical stability of reactive $O_{2}^{-}$ responsible for oxidative stress.

Unfortunately, we had no equipment for properly characterizing the level of developed oxidative stress and we consider providing such data in future publications. As for the analysis of the impact of magnesium ions accumulation in the dispersion medium, which was discussed in the literature earlier [30,32], in the systems studied here, the number of ions remained on the level of physiological concentration of magnesium [45,49] and hardly can be considered as excessive.

One more point that should be discussed here is related to the bacterial cells repelling by superhydrophobic surfaces. As was discussed earlier [38,40], the water-repelling properties of superhydrophobic coatings combined with electrostatic repulsion between the negatively charged superhydrophobic surface and negatively charged bacterial cell’s membrane are responsible for the above phenomena. The inspection of images of the layer of mixed magnesium, sodium, and potassium phosphates formed on top of the textured superhydrophobic surfaces in the PBS dispersion did not indicate the presence of deposited bacterial cells. However, we did not find bacterial cells on top of the covering layer on the superhydrophilic surfaces either. Thus, we have no unambiguous indication of the peculiar properties of the superhydrophobic surface, which would be responsible for the absence of cells deposited onto the covering layer.

## 4. Conclusions

In this work, in order to elucidate the mechanisms of the bactericidal action of magnesium, we analyzed the change in the bacterial titer in dispersions in contact with magnesium alloy samples with different wettability. The data obtained made it possible to distinguish two mechanisms that, apparently, play a key role in the bacterial decontamination of the liquid. These are the alkalization of the dispersion medium and the collection of bacterial cells by microrods growing on the surface as a result of the interaction of magnesium with the components of the buffer solution. The kinetics of changes in the titer of live bacteria and pH indicated that for magnesium samples with different wettability, the antibacterial activity is described by a single scenario and a single master curve. In this case, the differences in antibacterial efficiency are associated with the effect of wetting on the corrosion processes on the surface. This is why the superhydrophobic samples, being corrosion resistant under conditions of microbiologically induced corrosion, demonstrated the lowest antibacterial efficiency at short contact times of the bacterial dispersion with magnesium substrates, whereas superhydrophilic ones demonstrated the highest. At the same time, superhydrophobic coatings turned out to be useful for solving another urgent problem associated with obtaining the coatings that lead to a prolonged effect of increasing the pH and suppressing the vital activity of bacteria, while preserving the sample from corrosion damage.

## Figures and Tables

**Table 1:** Typical contact (CA) and roll-off (RA) angles for different samples.

State of the Coating	Bare Sample	Superhydrophilic Sample	Superhydrophobic Sample		
			Gray Area		White Area
	CA, Deg	CA, Deg	CA, Deg	RA, Deg	CA, Deg
Freshly prepared coatings	59.0 ± 2.0	0 Complete droplet wicking	171.2 ± 0.7	3.3 ± 0.9	–
After 48 h of immersion in PBS	31 ± 10	0 Complete droplet wicking	163.3 ± 1.0	14.3 ± 3.9	20 ± 10 Hemi-wicking
After 48 h of immersion in PBS, followed by heating in an oven at 180 °C for 30 min	49 ± 10	10.0 ± 5 Hemi-wicking	170.7 ± 1.0	3.5 ± 2.0	53.0 ± 5
After 48 h of immersion in bacterial dispersions	38 ± 12	0 Complete droplet wicking	170.3 ± 1.5	4.9 ± 2.4	85 ± 10

## Data Availability

Data are contained within the article.

## Supplementary Materials

The following are available online at https://www.mdpi.com/article/10.3390/ma14185454/s1: Figure S1 and discussion: the evolution of the concentration of magnesium and phosphorus in liquid PBS without bacteria versus time of contact with polished bare, superhydrophilic, or superhydrophobic MA8 samples, and the elemental composition of the surface layers of samples after 48 h contact. Figure S2: survey SEM images of the surfaces of studied magnesium alloy samples with different wettability after immersion for 48 h into the bacterial dispersions in PBS. Figure S3: The high-magnification SEM images of K. pneumoniae and P. aeruginosa on the surface texture of superhydrophilic sample. Video S1: The behavior of the water droplets on top of superhydrophobic surfaces contacted with a bacterial dispersion: droplets sticking before and droplets roll-off after water jet washing.
