# Supplementary material for: The Mechanisms of Antibacterial Activity of Magnesium Alloys with Extreme Wettability
Source: Materials (Basel). 2021 Sep 21;14(18):5454. doi: 10.3390/ma14185454 (PMC8470035; doi:10.3390/ma14185454)
Supplement: Supplementary file 1 [file materials-14-05454-s001.zip › materials-1316821-supplementary-XML.pdf]

materials

MDPI

Supplementary Materials

The Mechanisms of Antibacterial Activity of the Magnesium Alloy with Extreme Wettability

Alexandre M. Emelyanenko [1], Alexander G. Domantovsky [1], Valery V. Kaminsky [2,3], Ivan S. Pytskii [1], Kirill A. Emelyanenko [1] and Ludmila B. Boinovich [1,*]

[1] A.N. Frumkin Institute of Physical Chemistry and Electrochemistry, Leninsky Prospect 31 Bldg. 4, 119071 Moscow, Russia; ame@phyche.ac.ru (A.M.E.); doman-alex@yandex.ru (A.G.D.); ivanpic4586@gmail.com (I.S.P.); emelyanenko.kirill@gmail.com (K.A.E.)
[2] G.N. Gabrichevsky Research Institute for Epidemiology and Microbiology, 10 Admiral Makarov St., 125212 Moscow, Russia; kaminskyvalery86@gmail.com
[3] Russian Scientific Center of Roentgen Radiology, Ministry of Healthcare of the Russian Federation, 117997 Moscow, Russia
* Correspondence: boinovich@mail.ru; Tel.: +7-495-955-4625

S1. The Evolution of the Concentration of Magnesium and Phosphorus Obtained in Liq-Uid Pbs Without Bacteria

The evolution of the concentration of magnesium and phosphorus obtained in liquid PBS without bacteria is presented in Figure S1a for three cups containing polished bare, superhydrophilic, or superhydrophobic MA8 samples. The ratio of the metal sample apparent surface area to PBS volume was kept equal to 0.8 cm^2/mL in each cup. The non-monotonic behavior of magnesium concentration in the liquid phase is associated with the formation of covering layer for the bare sample and of covering layer and microrods for the superhydrophilic and superhydrophobic samples (Figure 2 of the main manuscript). The analysis of the elemental composition of the covering layer revealed the impact of the sample wettability on oxygen content (Figure S1b, black, green, and blue lines for the bare, superhydrophilic, and superhydrophobic samples, respectively). In contrast, the composition of rods was similar for samples with different wettability (Figure S1b, red line). It is worth noting that covering layers for all samples contacted to bacteria-free PBS did not accumulate sodium or potassium. At the same time, the rods, formed on top of superhydrophilic and superhydrophobic samples captured an ample amount of these alkali metals from PBS.

Corrosion induces rapid dissolution of the magnesium and growth of the Mg^{2+} ions concentration in the cups with superhydrophilic and bare samples. Chemical interaction of magnesium ions with phosphate-containing ions leads to the continuous growth of covering layer and rods, which, in turn, is accompanied by the dropping down of phosphorus and magnesium concentration in the liquid medium. Some inhibition of the growth of covering layer and crystal rods after 12 h of contact of PBS and metal samples is explained by depletion of the phosphorus-containing ions in the solution, and hence, the deceleration of their mass transfer to the front of growing new phase. From 12 to 24 h, the continuation of the corrosion dissolution of magnesium resulted in an increase in Mg^{2+} content in the liquid medium at the simultaneous slow decrease in the concentration of phosphorus, with a maximal concentration of magnesium reached after 24 h. At the same time, the layer of weakly soluble reaction products accumulated on the metal surface (Figure 2) acts as a barrier for ions and water molecules transfer and thus inhibits corrosion dissolution. The above concurrent processes lead to some decrease in the concentration of Mg^{2+} ions in a liquid. It is worth noting that bare polished MA8 contacted to PBS caused a higher concentration of Mg^{2+} ions in comparison to that established in PBS contacted to a superhydrophilic sample. Seemingly, such difference is related to the fact that in the latter

case magnesium ions in liquid medium were consumed to feed the growth of densely seeded hydrophosphate rods, while on bare substrates such rods were not formed.

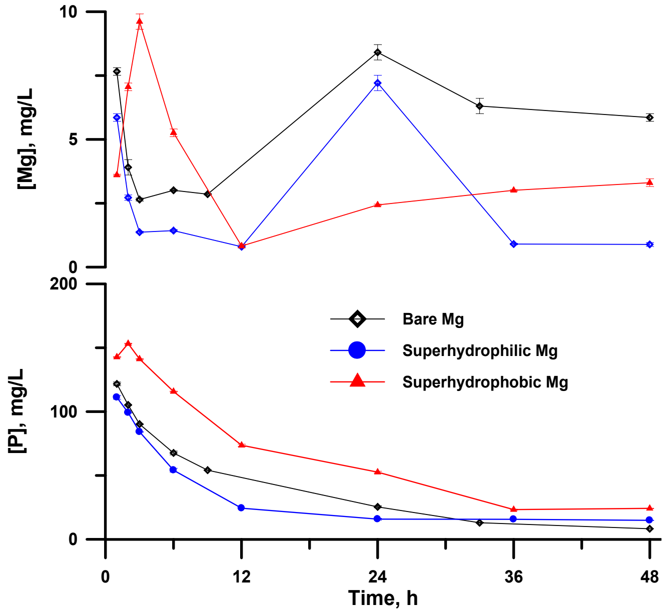

(**a**)

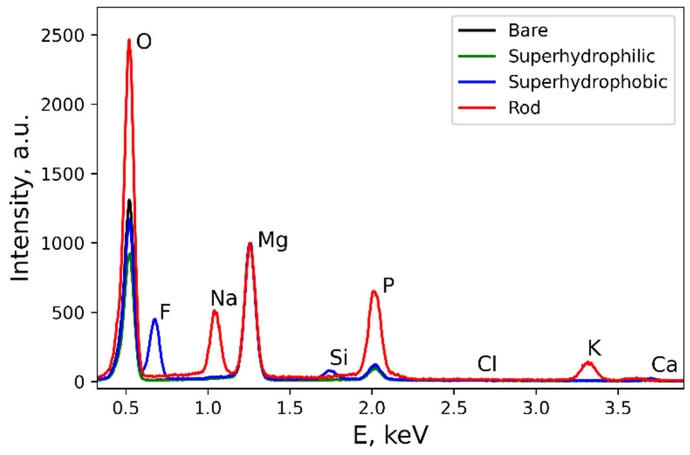

(**b**)

Figure S1. Variation in composition of various components of the system during prolonged contact between the PBS and MA8 magnesium alloy samples with different wettability. (**a**) Time evolution of magnesium and phosphorus content in PBS; (**b**) EDS spectra illustrating the elemental composition of the covering layer and rods formed on the surface of metal samples with different wettability after 48 h of contact with phosphate buffer solution. In the legend, "Rod" denotes the spectrum registered from the rod surface.

S2. Survey SEM Images of the Surfaces of Studied Samples.

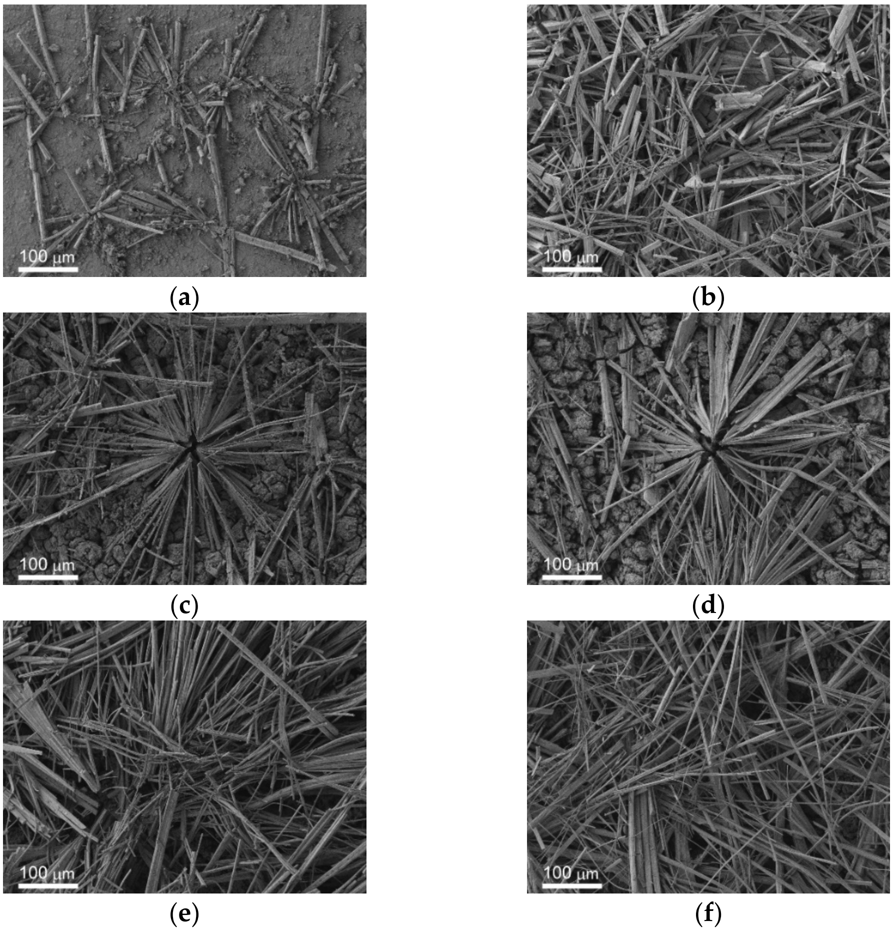

Figure S2. The survey SEM images of the surfaces of studied magnesium alloy samples with different wettability after immersion for 48 h into the bacterial dispersions in PBS. (**a**,**b**) – Polished bare MA8 sample after immersion into the dispersion of *K. pneumoniae* (**a**) and *P. aeruginosa* (**b**); (**c**,**d**)– Superhydrophilic sample after immersion into the dispersion of *K. pneumoniae* (**c**) and *P. aeruginosa* (**d**); (**e**,**f**) – Superhydrophobic sample after immersion into the dispersion of *K. pneumoniae* (**e**) and *P. aeruginosa* (**f**).

S3. SEM images of bacteria on the surfaces of studied samples.

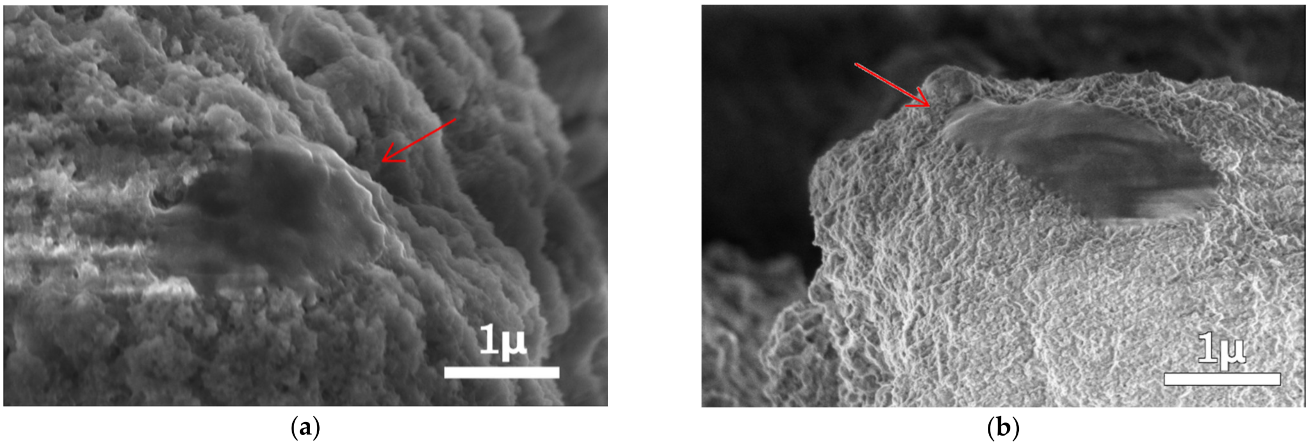

Figure S3. The high-magnification SEM images of *K. pneumoniae* (**a**) and *P. aeruginosa* (**b**) on the surface texture of superhydrophilic sample after 2 h of contact with the bacterial dispersion.
